# Selection and validation of reference genes for normalization of quantitative real-time reverse transcription PCR analysis in *Poria cocos* (Schw.) Wolf (*Fuling*)

**DOI:** 10.1186/s13020-016-0079-8

**Published:** 2016-03-02

**Authors:** Xin Zhang, Zhi-Chao Xu, Jiang Xu, Ai-Jia Ji, Hong-Mei Luo, Jing-Yuan Song, Chao Sun, Yuan-Lei Hu, Shi-Lin Chen

**Affiliations:** Institute of Medicinal Plant Development, Chinese Academy of Medical Sciences and Peking Union Medical College, Beijing, 100193 China; Institute of Chinese Materia Medica, China Academy of Chinese Medical Sciences, Beijing, 100700 China; Chongqing Institute of Medicinal Plant Cultivation, Chongqing, 408435 China; State Key Laboratory of Protein and Plant Gene Research, College of Life Sciences, Peking University, Beijing, 100871 China

## Abstract

**Background:**

Quantitative real-time reverse transcription PCR (qRT-PCR) requires a stable internal control to avoid misinterpretation of data or errors for gene expression normalization. However, there are still no validated reference genes for stable internal control in *Poria cocos* (Schw.) Wolf (*Fuling*). This study aims to validate the reference genes of *P. cocos*.

**Methods:**

This study firstly collected the 14 candidate reference genes by BLASTP from the genome of *P. cocos* for qRT-PCR analysis to determine the expression levels of 14 housekeeping genes (*GAPDH*, *MAPK*, *β*-*Act*, *RPB2*, *RPB1*-*1*, *RPB1*-*2*, *his3*-*1*, *his3*-*2*, *APT*, *SAMDC*, *RP*, *β*-*Tub*, *EIF*, and *CYP*) under different temperatures and in response to different plant hormones (indole-3-acetic acid, abscisic acid, 6-benzylaminopurine, methyl jasmonate, and gibberellic acid), and the threshold cycle (C_t_) values. The results were analyzed by four programs (i.e., geNorm, NormFinder, BestKeeper, and RefFinder) for evaluating the candidate reference genes.

**Results:**

*SAMDC*, *his3*-*2*, *RP*, *RPB2*, and *his3*-*1* were recommended as reference genes for treating *P. cocos* with indole-3-acetic acid, abscisic acid, 6-benzylaminopurine, methyl jasmonate, and gibberellic acid, respectively. Under different temperatures *RPB2* was the most stable reference gene. *CYP* was the most stable gene for all 90 samples by RefFinder.

**Conclusion:**

*SAMDC*, *his3*-*2*, *RP*, *RPB2*, and *his3*-*1* were evaluated to be suitable reference genes for *P. cocos* following different treatments. *RPB2* was the most stable reference gene under different temperatures and *CYP* was the most stable gene in the mycelia under all six evaluated conditions.

## Background

Quantitative real-time reverse transcription PCR (qRT-PCR) is used for determining the abundance of mRNAs in molecular biology studies. Suitable reference genes are necessary to ensure accuracy and to avoid bias. Typically, reference genes are housekeeping genes necessary for cellular metabolism. The genes for cyclophilin (*CYP*), tubulin, ubiquitin, glyceraldehyde-3-phosphate dehydrogenase (*GAPDH*), actin, 18S ribosomal RNA, 28S ribosomal RNA, and albumin are among the most frequently used reference genes [1].

However, the expression levels of reference genes may not be stable in different species [2], different tissues [3], or even identical cells under different culture conditions [4]. For example, the biosynthesis of triterpenes was induced by methyl jasmonate (MeJA) in *Ganoderma lucidum* (Leyss. ex Fr.) P. Karst (*Lingzhi*) [5, 6]. However, the stability of fungal reference genes in the presence of plant hormones has not been properly evaluated by the gene expression levels of enzymes involved in the triterpene biosynthesis pathway.

Little research has been conducted on reference genes in fungi. In *Hemileia vastatrix* Berk. and Br. (*Toubaoxiujun*), the cytochrome *b*, 40S ribosomal protein and *Hv00099* genes have been selected as reference genes in vitro; however, the 40S ribosomal protein, *GAPDH*, and *Hv00099* genes were the most stable genes *in planta* [7]. In *Hypocrea jecorina* Berk. and Br. (*Hongherouzuojun*), the gene encoding a GTPase was recommended as a reference gene [8]. Reference genes for qRT-PCR under different culture conditions and at different developmental stages in *G. lucidum* were reported [9].

*Poria cocos* (Schw.) Wolf (*Fuling*) is medicinal fungi and nutrition food widely distributes in East Asia, particularly in China, North America, Africa, and Australia [10, 11]. Pharmaceutically active constituents extracted from *P*. *cocos*, including polysaccharides, triterpene derivatives, lanostane derivatives, and poricoic acid, exhibited anti-oxidant [12, 13], anti-inflammatory [14], anti-tumor [15–17], anti-emetic [18], anti-nephritic [19], anti-rejection [20], diuretic [21], and anti-hyperglycemic activities [22]. The nematicidal activity of *P. cocos* was investigated and the active compounds were isolated [23]. Studies on the molecular biology of *P*. *cocos* were limited, including the basic molecular studies such as gene expression analysis and gene function identification [24]. qRT-PCR method was effective to detect the candidate genes involved in secondary metabolite biosynthesis. For example, the genes are most likely involved in the biosynthesis of pachymic acid in *P. cocos* was identified by qRT-PCR [25]; however, contigs and singletons were used instead of reference genes. The stability of potential internal control genes in *P. cocos* has not been evaluated.

This study aims to discover and obtain the stable reference genes of *P. cocos* for normalization of qRT-PCR analysis.

## Methods

### Sampling and culture conditions

The *P. cocos* strain CGMCC5.78 was purchased from the Institute of Microbiology, Chinese Academy of Sciences and was stored in the Institute of Medicinal Plant Development at −80 °C. We identified the strain using the DNA barcoding method with *ITS2* primers. Ninety mycelial samples under different culture conditions were used in this study. Vegetative mycelia were cultured in two different media: potato dextrose agar medium (AOBOX, Beijing, China) and sucrose medium. The components of the sucrose medium were as follows: vitamin B1, 0.05 g/L; MgSO_4_∙7H_2_O, 0.5 g/L; KH_2_PO_4_∙H_2_O, 1 g/L; yeast extract, 2.5 g/L; peptone, 5 g/L; and sucrose, 35 g/L. The strain was maintained in potato dextrose agar medium. In the preculture stage, 40-mL sucrose medium was inoculated with mycelia and shaken (Thermo Fisher Scientific 491, Waltham, MA, USA) at 50 rpm in the dark in an incubator at 28 °C for 1 week. Subsequently, all of the mycelia were spread and were shaken at 120 rpm for an additional week in the dark at 28 °C. Finally, all cultures, including the culture broth, were incubated under various conditions (Table 1), including different concentration of hormones and different temperatures for 24 h.

**Table 1:** Different treatment conditions applied to the mycelia of *P. cocos*

Treatment	Group	Treatment conditions				
IAA (mg/L)	A	10	20	30	40	50
ABA (mg/L)	B	10	20	30	40	50
6-BA (mg/L)	C	0.01	0.1	1	5	10
MeJA (μm/L)	D	5	10	50	100	200
GA (mg/L)	E	10	20	30	40	50
Temperature (°C)	F	4	15	20	28	40

The samples were arbitrarily allocated into six groups for analysis (Table 1). The samples in groups A, B, C, D, and E were cultured in the media supplemented with different concentrations of indole-3-acetic acid (IAA; Sangon, Shanghai, China), abscisic acid (ABA; Sangon), 6-benzylaminopurine (6-BA;Sigma, St Louis, MO, USA), methyl jasmonate (MeJA; Sigma), and gibberellic acid (GA; Sangon), respectively. Group F comprised samples collected from cultures incubated at five different temperatures. The mycelia were collected by double gauze filters (CWBio, Beijing, China). Each experiment was performed in triplicate. A total of 90 samples were collected, and all of the samples were frozen in liquid nitrogen and stored at −80 °C.

### Total RNA extraction, DNase treatment, and cDNA synthesis

The liquid nitrogen frozen samples were ground into fine powder by a mortar and pestle. The total RNA of each sample was extracted by the Polysaccharide and Polyphenol Total RNA Isolation Kit (spin column; BioTeke, Beijing, China) according to the manufacturer’s instructions. The total RNA integrity and quality were confirmed by 1 % agarose gel electrophoresis by ethidium bromide staining. The RNA concentration was determined by a NanoDrop2000 spectrophotometer (Thermo Fisher Scientific, Waltham, MA, USA). One microgram of total RNA of each sample was reverse transcribed by the FastQuant RT Kit (with gDNase; TIANGEN, Beijing, China) according to the manufacturer’s protocol. All templates were diluted 30-fold for PCR and qRT-PCR.

### Candidate gene selection, primer design, and validation

Based on previous studies [1, 3, 4] of reference genes determined in other species, 14 genes were evaluated in the present study, including multiple-copy genes. These genes include glyceraldehyde-3-phosphate dehydrogenase (*GAPDH*), mitogen-activated protein kinase (*MAPK*), beta actin (*β*-*Act*), RNA polymerase subunit2 (*RPB2*), RNA polymerase subunit1 (*RPB1*), histone 3 (*his3*), adenine phosphoribosyl transferase (*APT*), *S*-adenosyl methionine decarboxylase (*SAMDC*), ribosomal protein (*RP*), beta tubulin (*β*-*Tub*), eukaryotic translation initiation factor (*EIF*), and cyclophilin (*CYP*). The primer sequences, amplicon size and number of gene copies in the genome are summarized in Table 2. The candidate genes were selected from the *P. cocos* genome sequence database (SRA: PRJNA42921) by the BLASTP program (National Library of Medicine, USA) and a threshold *E*-value <1 × 10^−50^.

**Table 2:** Descriptions of the 14 candidate reference genes and their primer sequences for qRT-PCR

Gene	Gene description	Primer sequences (forward/reverse)	Amplicon length (bp)	Access number	Total copy numbers
*GAPDH*	Glyceraldehyde 3-phosphate dehydrogenase	TGTTCGTCTGCGGTGTCA/AGTGGACGGTGGTCATCAG	150	KJ716556	1
*MAPK*	Mitogen-activated protein kinase	CACATCCAGCACGAGAACAT/GGAGGATCTGGTAGAGGAAGTA	163	KJ716546	10
*β*-*Act*	Beta actin	ATGCGAGGTTATGCGTTCA/CCGACCATCTGGGAGTGTAT	156	KJ716554	2
*RPB2*	RNA polymerase subunit 2	ACCAACTTCCTCGTCAGAATG/TCCTGATTGTATCCGCTGTAAC	161	KJ716552	1
*RPB1*-*1*	RNA polymerase subunit 1	GGCTTACAACAGGTCGTCAA/CGTGGCGTCCTCAATAACTT	153	KJ716547	2
*RPB1*-*2*	RNA polymerase subunit 1	AGGATGACGAAGCAGAGGAA/TGGCATTGGGCAGGTTCT	168	KJ716548	
*his3*-*1*	Histone 3	AGTCCACGGAACTCCTAATCA/AGCGGCTAAGTTGGTGTCT	167	KJ716557	3
*his3*-*2*	Histone 3	CGACGGAGTTGCTCATCAG/GTGGATCGCAGCCAGATTC	170	KJ716544	
*APT*	Adenine phosphoribosyltransferase	ACCTGAGGAGTCTGCTGAAG/TTGTGGAATAGTGTGCGATGT	149	KJ716549	1
*SAMDC*	*S*-adenosylmethionine decarboxylase	GCTTCTACTCTCGCAAGGC/GATATACAGCAGCCAGTGGTC	155	KJ716550	1
*RP*	Ribosomal protein	TGTCGCTCTCCTCAAGTCC/CGGAATGCCTTGACGATACC	165	KJ716551	1
*β*-*Tub*	Beta tubulin	GCCAACATACGGTGATCTGAA/GAAGAAGTGAAGACGAGGGAAT	142	KJ716555	1
*EIF*	Eukaryotic translation initiation factor	TGACGATGACAGCGATGAAG/CACCTGGACTGCCTTATGC	145	KJ716545	1
*CYP*	Cyclophilin	CATGGCTTCGGCTACAAGG/TTGGTGTGCTTGAGCTTGAA	152	KJ716553	3

Primer Premier 6.0 (PREMIER Biosoft, USA) and DNAMAN (LynnonBiosoft, USA) were used for primer design with the following criteria: an amplicon size ranging from 130 to 180 bp, an optimal *T*_m_ of 53–55 °C, and a primer length from 18 to 22 bp. The primers were synthesized by Sangon Biotech (Shanghai, China). The specificity of each primer pair was measured by 2 % agarose gel electrophoresis following PCR (95 °C for 5 min; 35 cycles of 95 °C for 15 s and 60 °C for 1 min; 72 °C for 10 min) by the 90 cDNA sample mixture. Additionally, qRT-PCR was performed and the melting curve was determined for primers specific validation.

### Real-time PCR performance and C_t_ data collection

The expression level of each gene was determined in 96-well plates by an Applied Biosystems 7500 Real-Time PCR system (Life Technologies, Grand Island, NY, USA). Each reaction mixture contained 200 nM of each primer, 2 µL of the prepared cDNA template, 4.9-µL ddH_2_O, and 7.5-µL Ultra SYBR Mixture with ROX (CWBio, Beijing, China) in a final volume of 15 µL. The amplifications were performed by an initial denaturation step of 95 °C for 5 min, followed by 45 cycles of 95 °C for 15 s and 60 °C for 1 min. A temperature ramp step was added after 45 amplification cycles for specificity analysis (melting curve), with 95 °C for 15 s, 60 °C for 1 min, 95 °C for 15 s, and a final temperature of 60 °C for 15 s. There were three biological duplicate samples, and each biological duplicate sample was evaluated in triplicate.

### Data analysis

The C_t_ values from each reaction were used for analysis of the expression levels of all detected reference genes. The geNorm [26], NormFinder [27], BestKeeper [28], the Delta CT method [29] and the Web-based tool RefFinder [30] were used to determine the stability of the candidate reference genes. The default parameters of these software were applied.

## Results

### Expression profile of candidate reference genes

The mean C_t_ value was computed by three biological duplicates and three technical replicates for each independent experiment (the template generated from each condition of *P. cocos* was used in different independent experiment), and the three technical replicates were performed independently. A higher C_t_ value indicates decreased transcription of the target gene. The average C_t_ value of each candidate gene under conditions ranged from 22.45 ± 0.97 to 32.86 ± 0.86 cycles (Table 3). The average C_t_ value of six of the 14 genes was higher than 30.00. *RPB1*-*2* and *CYP* demonstrated the lowest and highest relative expression levels, with average C_t_ values of 31.21–33.21 and 22.37–23.91, respectively. The variation in the C_t_ value was determined by the maximum and minimum C_t_ values. The variation in the C_t_ value of each candidate reference gene in all 90 samples ranged between 3.22 and 7.89. *RPB1*-*1* exhibited the lowest variation in C_t_ value followed by *CYP* (3.24). In contrast, *EIF* exhibited the highest variation in C_t_ value (7.89).

**Table 3:** The average C_t_ value (mean ± SD) of each candidate gene under different conditions

	*GAPDH*	*MAPK*	*β*-*Act*	*RPB2*	*RPB1*-*1*	*RPB1*-*2*	*his3*-*1*	*his3*-*2*	*APT*	*SAMDC*	*RP*	*β*-*Tub*	*EIF*	*CYP*
IAA	22.88 ± 1.26	29.49 ± 0.94	30.13 ± 0.83	29.60 ± 1.30	31.31 ± 1.19	32.10 ± 0.97	26.49 ± 1.06	25.53 ± 1.43	29.11 ± 1.05	32.57 ± 0.97	28.21 ± 0.80	27.98 ± 0.89	28.57 ± 0.96	22.45 ± 0.97
ABA	23.69 ± 0.73	30.76 ± 0.74	30.66 ± 1.50	29.71 ± 2.03	31.58 ± 1.34	31.83 ± 1.54	27.04 ± 0.82	26.22 ± 0.62	29.43 ± 0.88	32.44 ± 0.87	28.39 ± 0.70	28.18 ± 1.05	28.92 ± 0.66	23.35 ± 0.48
6-BA	22.66 ± 1.23	27.48 ± 0.81	31.00 ± 0.87	29.81 ± 1.06	31.57 ± 1.01	32.51 ± 1.27	26.53 ± 0.68	22.37 ± 0.92	32.06 ± 1.01	28.40 ± 1.19	27.96 ± 0.69	24.12 ± 1.05	24.51 ± 1.09	23.26 ± 0.74
MeJA	23.91 ± 1.23	30.85 ± 1.18	29.97 ± 0.78	32.45 ± 1.00	30.69 ± 0.81	32.86 ± 0.86	26.91 ± 0.91	26.73 ± 0.86	31.65 ± 0.95	33.24 ± 1.18	28.20 ± 1.15	28.13 ± 1.23	29.95 ± 0.92	23.53 ± 0.67
GA	24.33 ± 1.32	31.47 ± 0.61	30.36 ± 1.28	31.82 ± 1.26	30.60 ± 1.20	32.06 ± 0.96	26.52 ± 1.23	25.66 ± 1.27	31.48 ± 1.34	32.58 ± 1.22	27.48 ± 1.61	27.72 ± 1.64	29.22 ± 1.53	23.48 ± 0.96
Temperature	23.16 ± 1.01	27.08 ± 1.18	30.37 ± 0.68	29.94 ± 0.88	31.50 ± 0.93	31.90 ± 0.94	25.87 ± 1.56	22.30 ± 0.77	32.12 ± 1.08	28.64 ± 1.17	27.37 ± 1.77	24.27 ± 1.11	25.08 ± 1.04	22.80 ± 0.93

### Stability ranking of candidate reference genes

**Fig. 1:**
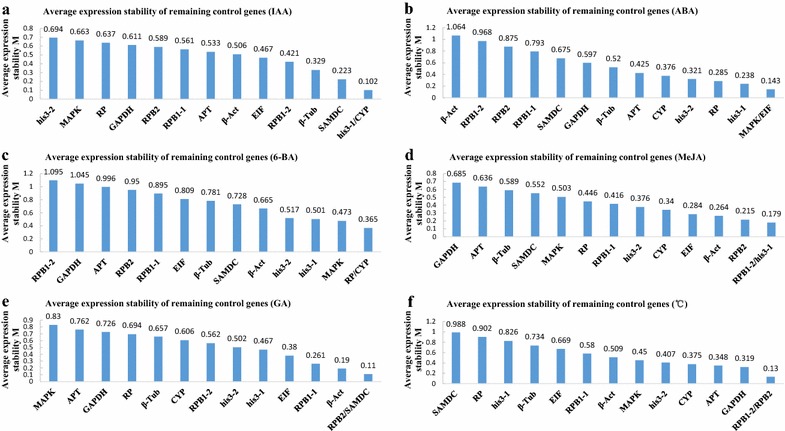
Stability values of the candidate reference genes calculated using geNorm under different treatment conditions. **a**, **b**, **c**, **d**, and **e** Stability values of the 14 candidate genes treated with IAA, ABA, 6-BA, MeJA, and GA, respectively. **f** Stability values of the14 candidate genes under different temperatures

**Fig. 2:**
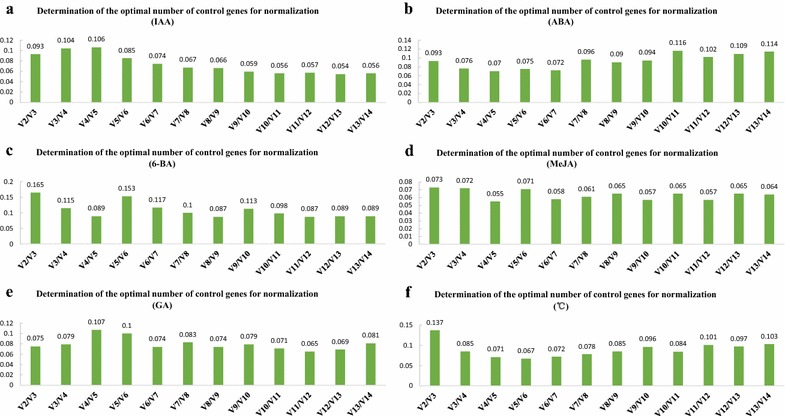
Determination of the optimal number of control genes for normalization under different treatment conditions. If the *V*_2_/*V*_3_ value is less than 0.15, two genes are suitable for normalization as recommended by geNorm. Otherwise, more genes are needed until the *V*_*n*_/*V*_*n*+1_ value is less than 0.15, and the suitable gene number for normalization is *n*. **a**, **b**, **c**, **d**, and **e** Optimal number of control genes for normalization after IAA, ABA, 6-BA, MeJA, and GA treatment, respectively. **f** Optimal number of control genes for normalization under different temperatures

**Fig. 3:**
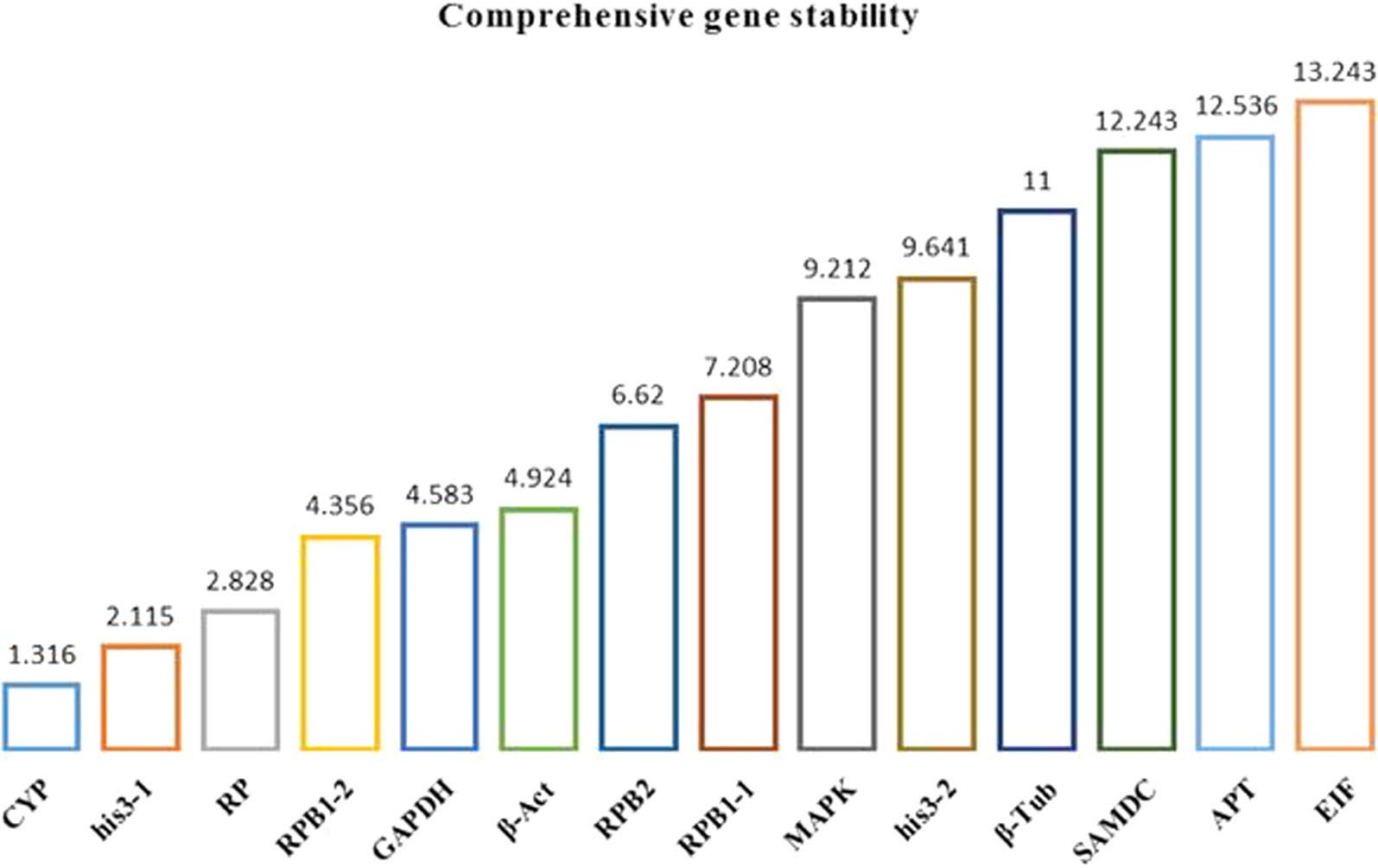
Comprehensive gene stability. Ranking order of gene stability in all samples under the various conditions using Delta CT, geNorm, NormFinder, and BestKeeper

geNorm ranks the potential reference genes on the basis of their average pairwise variation in expression of one gene compared with each other gene of the set [26]. geNorm recommends 1.5 as the M-value cutoff. An M-value of less than 1.5 indicates stable expression, with the lowest M-value corresponding to the highest stability, and *vice versa*. Two reference genes were recommended for an ideal relative quantitative analysis. The M-values of candidate genes under different conditions generated by geNorm are listed in Table 4. The stability of the genes under different treatment conditions analyzed by geNorm is shown in Figs. 1, 2, 3. In group A, *his3*-*1* and *CYP* were the most stable genes, and *his3*-*2* was the most unstable gene. In group B, *MAPK* and *EIF* exhibited the highest stability, and *β*-*Act* exhibited the lowest stability. Under different temperatures, the expression levels of *RPB1*-*2* and *RPB2* were the most stable in the cultured mycelia, and *SAMDC* exhibited a performance that was worse than those of the other 13 genes. When treated with different concentrations of 6-BA, *RP* and *CYP* were the most stable reference genes, and *RPB1*-*2* exhibited the highest M-value. Following treatment with MeJA, an inducer of secondary metabolism [5, 6], the best reference genes were *RPB1*-*2* and *his3*-*1*, whereas *GAPDH* was the most unstable. Following GA treatment, the expression level of *MAPK* exhibited the most variation, whereas *RPB2* and *SAMDC* were considered suitable reference genes. Following treatment with IAA, ABA, different temperatures, 6-BA, MeJA, and GA, the average M-values were 0.517–0.871, 0.768–1.36, 0.734–1.242, 0.857–1.333, 0.521–0.849, and 0.602–1.058, respectively. These values suggested that 6-BA contributed the most to the variation in expression levels of the reference genes. A geNorm analysis using all samples indicated that *his3*-*1* and *RP* were the most stable genes with the lowest M-values, and *APT* was the most variable gene.

**Table 4:** The M value of each candidate gene under different conditions generated by geNorm

	*GAPDH*	*MAPK*	*β*-*Act*	*RPB2*	*RPB1*-*1*	*RPB1*-*2*	*his3*-*1*	*his3*-*2*	*APT*	*SAMDC*	*RP*	*β*-*Tub*	*EIF*	*CYP*
IAA	0.745	0.845	0.637	0.732	0.730	0.698	0.556	0.879	0.668	0.508	0.824	0.644	0.704	0.546
ABA	0.984	0.809	1.644	1.324	1.274	1.583	0.932	0.784	0.842	1.045	0.853	1.189	0.789	0.848
6-BA	1.352	0.971	1.032	1.178	1.120	1.392	1.023	1.022	1.290	0.985	0.798	1.125	1.177	0.861
MeJA	0.981	0.766	0.537	0.506	0.715	0.520	0.554	0.592	0.937	0.793	0.686	0.842	0.551	0.617
GA	0.950	1.237	0.707	0.716	0.691	0.872	0.658	0.703	1.007	0.732	0.934	0.891	0.730	0.795
Temperature	0.857	0.887	0.852	0.730	0.964	0.753	1.286	0.750	0.808	1.057	1.414	1.132	1.135	0.758

NormFinder is an Excel-based program for evaluating the expression stability of candidate reference genes based on the expression values, which enables estimation not only of the overall variation of the candidate normalization genes but also of variation between sample subgroups of the sample set [27]. NormFinder shows less sensitivity toward coregulation of the candidate normalization genes. A lower stability value indicates a higher stability. In group A, *SAMDC* was the most stable gene, with a stability value of 0.135, whereas *his3*-*2* was the most unstable gene, with a stability value of 0.769. In group B, *his3*-*2* exhibited the lowest stability value of 0.088, and *β*-*Act* exhibited the highest stability value of 1.586. Under different temperatures, *his3*-*2* was the most stable, with a stability value of 0.069, and *SAMDC* was the least stable, with a stability value of 1.428. In group C, *RP* exhibited the best performance with a stability value of 0.183, and the expression level of *RPB1*-*2* varied the most under different concentrations of 6-BA, with a stability value of 1.228. Following MeJA treatment, *RPB2* exhibited the lowest variation, with a stability value of 0.106, and *GAPDH* exhibited the lowest stability value of 0.887. In group E, *his3*-*1* was recommended as the reference gene for GA treatment, with a stability value of 0.244, and *MAPK* was the most unstable gene among the 14 genes, with a stability value of 1.119. When all of the samples were analyzed, *CYP* exhibited the lowest stability value of 1.320, whereas *EIF* exhibited the highest stability value at 13.240.

Gene expression stability was evaluated by BestKeeper using the standard deviation (SD), percentage covariance (CV), and correlation coefficient (*r*) [28]. BestKeeper can determine the best suited standards, out of 10 candidates, and combine them into an index. The candidate reference genes with SD >1 are considered unstable, and a higher SD value indicates greater variation. From groups A to F, the recommended reference genes were *β*-*Tub*, *CYP*, *RP*, *his3*-*2*, *MAPK*, and *EIF*, with SD values of 0.397, 0.171, 0.234, 0.420, 0.345, and 0.297, respectively. In addition, the genes exhibiting the highest SD values in groups A, B, C, D, E, and F were *his3*-*2* (SD = 1.115), *RPB1*-*2* (SD = 1.137), *GAPDH* (SD = 0.842), *MAPK* (SD = 0.949), *RP* (SD = 1.468), and *RP* (SD = 1.491), respectively. When all 90 samples were considered, the expression level of *CYP* was the most stable, whereas *EIF* was the most unstable, with SD values of 0.608 and 1.980, respectively.

RefFinder analysis integrates four different methods (i.e., Delta CT, geNorm, NormFinder, and BestKeeper). The C_t_ values were input into RefFinder directly, and the ranking of the four methods was calculated. Based on the rankings from each method, RefFinder assigns an appropriate weight to an individual gene and calculates the geometric mean of their weights for the overall final ranking [30]. The rankings of the candidate reference genes used in Delta CT were according to the repeatability of the gene expression differences among the samples. The results analyzed by RefFinder are summarized in Tables 5, 6, 7, 8, 9, 10. In group A, *SAMDC* was recommended as the most stable reference gene. In group B, *his3*-*2* exhibited the best performance, whereas *his3*-*1* exhibited the best performance in group E. Under different treatment temperatures and different concentrations of MeJA, *RPB2* maintained a stable expression level. Following 6-BA treatment, *RP* ranked as a suitable reference gene (Table 7). Following comprehensive analysis of all of the samples under the various conditions by Delta CT, geNorm, NormFinder, and BestKeeper, *CYP* was recommended as the reference gene.

**Table 5:** Ranking order of the reference genes with IAA treatment (Better-Good-Average)

Method	1	2	3	4	5	6	7	8	9	10	11	12	13	14
DeltaCT	*SAMDC*	*CYP*	*his3*-*1*	*β*-*Act*	*β*-*Tub*	*APT*	*RPB1*-*2*	*EIF*	*RPB1*-*1*	*RPB2*	*GAPDH*	*RP*	*MAPK*	*his3*-*2*
BestKeeper	*β*-*Tub*	*RP*	*RPB1*-*2*	*EIF*	*SAMDC*	*β*-*Act*	*CYP*	*MAPK*	*his3*-*1*	*RPB2*	*APT*	*RPB1*-*1*	*GAPDH*	*his3*-*2*
NormFinder	*SAMDC*	*CYP*	*his3*-*1*	*β*-*Act*	*β*-*Tub*	*APT*	*EIF*	*RPB1*-*2*	*RPB2*-*3*	*RPB1*-*1*	*GAPDH*	*RP*	*MAPK*	*his3*-*2*
geNorm	*his3*-*1/CYP*		*SAMDC*	*β*-*Tub*	*RPB1*-*2*	*EIF*	*β*-*Act*	*APT*	*RPB1*-*1*	*RPB2*	*GAPDH*	*RP*	*MAPK*	*his3*-*2*
Recommended comprehensive ranking	*SAMDC*	*CYP*	*his3*-*1*	*β*-*Tub*	*β*-*Act*	*RPB1*-*2*	*EIF*	*APT*	*RP*	*RPB2*	*RPB1*-*1*	*GAPDH*	*MAPK*	*his3*-*2*

**Table 6:** Ranking order of the reference genes with ABA treatment (Better-Good-Average)

Method	1	2	3	4	5	6	7	8	9	10	11	12	13	14
DeltaCT	*his3*-*2*	*EIF*	*MAPK*	*APT*	*CYP*	*RP*	*his3*-*1*	*GAPDH*	*SAMDC*	*β*-*Tub*	*RPB1*-*1*	*RPB2*	*RPB1*-*2*	*β*-*Act*
BestKeeper	*CYP*	*his3*-*2*	*RP*	*MAPK*	*EIF*	*his3*-*1*	*GAPDH*	*APT*	*β*-*Tub*	*SAMDC*	*RPB1*-*1*	*β*-*Act*	*RPB2*	*RPB1*-*2*
NormFinder	*his3*-*2*	*APT*	*EIF*	*CYP*	*MAPK*	*RP*	*GAPDH*	*his3*-*1*	*SAMDC*	*β*-*Tub*	*RPB1*-*1*	*RPB2*	*RPB1*-*2*	*β*-*Act*
geNorm	*MAPK/EIF*		*his3*-*1*	*RP*	*his3*-*2*	*CYP*	*APT*	*β*-*Tub*	*GAPDH*	*SAMDC*	*RPB1*-*1*	*RPB2*	*RPB1*-*2*	*β*-*Act*
Recommended comprehensive ranking	*his3*-*2*	*EIF*	*MAPK*	*CYP*	*RP*	*APT*	*his3*-*1*	*GAPDH*	*β*-*Tub*	*SAMDC*	*RPB1*-*1*	*RPB2*	*RPB1*-*2*	*β*-*Act*

**Table 7:** Ranking order of the reference genes with 6-BA treatment (Better-Good-Average)

Method	1	2	3	4	5	6	7	8	9	10	11	12	13	14
DeltaCT	*RP*	*CYP*	*MAPK*	*SAMDC*	*his3*-*2*	*his3*-*1*	*β*-*Act*	*RPB1*-*1*	*β*-*Tub*	*EIF*	*RPB2*	*APT*	*GAPDH*	*RPB1*-*2*
BestKeeper	*RP*	*CYP*	*SAMDC*	*his3*-*2*	*his3*-*1*	*β*-*Act*	*MAPK*	*RPB1*-*1*	*EIF*	*RPB2*	*β*-*Tub*	*APT*	*RPB1*-*2*	*GAPDH*
NormFinder	*RP*	*CYP*	*SAMDC*	*MAPK*	*β*-*Act*	*his3*-*2*	*his3*-*1*	*RPB1*-*1*	*β*-*Tub*	*RPB2*	*EIF*	*APT*	*GAPDH*	*RPB1*-*2*
geNorm	*RP/CYP*		*MAPK*	*his3*-*1*	*his3*-*2*	*β*-*Act*	*SAMDC*	*β*-*Tub*	*EIF*	*RPB1*-*1*	*RPB2*	*APT*	*GAPDH*	*RPB1*-*2*
Recommended comprehensive ranking	*RP*	*CYP*	*SAMDC*	*MAPK*	*his3*-*2*	*his3*-*1*	*β*-*Act*	*RPB1*-*1*	*β*-*Tub*	*EIF*	*RPB2*	*APT*	*GAPDH*	*RPB1*-*2*

**Table 8:** Ranking order of the reference genes with MeJA treatment (Better-Good-Average)

Method	1	2	3	4	5	6	7	8	9	10	11	12	13	14
DeltaCT	*RPB2*	*RPB1*-*2*	*β*-*Act*	*EIF*	*his3*-*1*	*his3*-*2*	*CYP*	*RP*	*RPB1*-*1*	*MAPK*	*SAMDC*	*β*-*Tub*	*APT*	*GAPDH*
BestKeeper	*his3*-*2*	*CYP*	*EIF*	*RPB1*-*1*	*RPB1*-*2*	*APT*	*RPB2*	*SAMDC*	*β*-*Act*	*his3*-*1*	*GAPDH*	*RP*	*β*-*Tub*	*MAPK*
NormFinder	*RPB2*	*β*-*Act*	*RPB1*-*2*	*EIF*	*his3*-*1*	*his3*-*2*	*CYP*	*RP*	*RPB1*-*1*	*MAPK*	*SAMDC*	*β*-*Tub*	*APT*	*GAPDH*
geNorm	*RPB1*-*2/his3*-*1*		*RPB2*	*β*-*Act*	*EIF*	*CYP*	*his3*-*2*	*RPB1*-*1*	*RP*	*MAPK*	*SAMDC*	*β*-*Tub*	*APT*	*GAPDH*
Recommended comprehensive ranking	*RPB2*	*RPB1*-*2*	*β*-*Act*	*EIF*	*his3*-*1*	*his3*-*2*	*CYP*	*RPB1*-*1*	*RP*	*SAMDC*	*APT*	*MAPK*	*β*-*Tub*	*GAPDH*

**Table 9:** Ranking order of the reference genes with GA treatment (Better-Good-Average)

Method	1	2	3	4	5	6	7	8	9	10	11	12	13	14
DeltaCT	*his3*-*1*	*RPB1*-*1*	*his3*-*2*	*β*-*Act*	*RPB2*	*EIF*	*SAMDC*	*CYP*	*RPB1*-*2*	*β*-*Tub*	*RP*	*GAPDH*	*APT*	*MAPK*
BestKeeper	*MAPK*	*RPB1*-*2*	*CYP*	*RPB1*-*1*	*RPB2*	*SAMDC*	*GAPDH*	*APT*	*his3*-*2*	*his3*-*1*	*β*-*Act*	*EIF*	*β*-*Tub*	*RP*
NormFinder	*his3*-*1*	*his3*-*2*	*RPB1*-*1*	*EIF*	*β*-*Act*	*RPB2*	*CYP*	*SAMDC*	*RPB1*-*2*	*β*-*Tub*	*GAPDH*	*RP*	*APT*	*MAPK*
geNorm	*RPB2/SAMDC*		*β*-*Act*	*RPB1*-*1*	*EIF*	*his3*-*1*	*his3*-*2*	*RPB1*-*2*	*CYP*	*β*-*Tub*	*RP*	*GAPDH*	*APT*	*MAPK*
Recommended comprehensive ranking	*his3*-*1*	*RPB1*-*1*	*RPB2*	*SAMDC*	*his3*-*2*	*β*-*Act*	*RPB1*-*2*	*EIF*	*CYP*	*MAPK*	*GAPDH*	*β*-*Tub*	*APT*	*RP*

**Table 10:** Ranking order of the reference genes under different temperatures (Better-Good-Average)

Method	1	2	3	4	5	6	7	8	9	10	11	12	13	14
DeltaCT	*RPB2*	*his3*-*2*	*RPB1*-*2*	*CYP*	*APT*	*β*-*Act*	*GAPDH*	*MAPK*	*RPB1*-*1*	*β*-*Tub*	*EIF*	*his3*-*1*	*RP*	*SAMDC*
BestKeeper	*EIF*	*β*-*Act*	*RPB2*	*RPB1*-*1*	*RPB1*-*2*	*β*-*Tub*	*his3*-*2*	*SAMDC*	*GAPDH*	*CYP*	*APT*	*MAPK*	*his3*-*1*	*RP*
NormFinder	*his3*-*2*	*RPB2*	*CYP*	*RPB1*-*2*	*APT*	*β*-*Act*	*GAPDH*	*MAPK*	*RPB1*-*1*	*β*-*Tub*	*EIF*	*his3*-*1*	*RP*	*SAMDC*
geNorm	*RPB1*-*2/RPB2*		*GAPDH*	*APT*	*CYP*	*his3*-*2*	*MAPK*	*β*-*Act*	*RPB1*-*1*	*EIF*	*β*-*Tub*	*his3*-*1*	*RP*	*SAMDC*
Recommended comprehensive ranking	*RPB2*	*RPB1*-*2*	*his3*-*2*	*β*-*Act*	*CYP*	*APT*	*EIF*	*GAPDH*	*RPB1*-*1*	*MAPK*	*β*-*Tub*	*SAMDC*	*his3*-*1*	*RP*

The results obtained using these different methods were not identical. In group C, *RP* was recommended as the most stable gene by all of these methods, whereas in group F, *RPB2* was recommended as the reference gene by Delta CT, geNorm and RefFinder. However, in the remaining groups, Delta CT, NormFinder and RefFinder recommended the same gene as the reference gene; *SAMDC*, *his3*-*2*, *RPB2* and *his3*-*1* in groups A, B, D, and E, respectively. Following comprehensive analysis of all of the samples under the various conditions, *CYP* was recommended as the reference gene by Delta CT, BestKeeper, NormFinder, and RefFinder, although not with geNorm. According to the above-mentioned results, RefFinder was likely the most comprehensive and scientific of these methods.

### Evaluation of the combination of reference genes

Pairwise variation (*V*) determines the optimal number of control genes for normalization and proposes 0.15 as a cutoff value [26]. If the *V*_*n*_/*V*_*n*+1_ value is less than 0.15, the suitable gene number for normalization is *n*. Additional control genes were not necessary in the six groups except for group C (i.e., the 6-BA treatment group),as indicated by *V*_2_/*V*_3_ values below 0.15 [26]. Three reference genes were recommended for group C, as indicated by a *V*_3_/*V*_4_ value of 0.115, which is consistent with the M-value ranking for this group.

## Discussion

Validation of the stability of candidate reference genes under different experimental conditions [31], with different tissues [32, 33], at different stages, and in different species [34] is necessary. In the present study, *EIF* was the most unstable gene in *P. cocos*; however, *EIF1* and *EIF3* were recommended as reference genes in *Ammopiptanthus mongolicus* (Maxim. ex Kom.) S.H. Cheng [35]. In contrast, *CYP* was the most stable gene in leaves of *Deschampsia antarctica* É. Desv. [36] under three abiotic stresses (salt, cold, and PEG treatment), whereas the *EF*-*1α* gene was recommended for roots. In banana fruit, the expression levels of two widely used reference genes, actin and *GAPDH*, were not stable [34].

The candidate reference gene rankings for the individual groups evaluated in this study may differ slightly from the ranking for all samples because, under specific circumstances, more accurate rankings would be established. Moreover, most of the M-values of the 14 genes were less than 1.5 except for *SAMDC*, *EIF*, and *APT*, indicating that most of the candidate reference genes were stable. As one of the least stable genes, the instability of *APT* has been reported in papaya under six experimental conditions [37]. It was contradictory that *CYP* was the best overall reference gene but did not exhibit the best performance in any single group. *CYP* was the most stable reference gene using Delta CT, NormFinder, and BestKeeper but not geNorm (Table 11). In addition, *CYP* was the third-most stable reference gene by geNorm. Moreover, *CYP* frequently ranked among the top five reference genes (Tables 5, 6, 7, 8, 9, 10), particularly under 6-BA treatment, in which *CYP* exhibited the highest average M-value when using geNorm for analysis. In group C, *CYP* ranked firmly as the second-most stable reference gene. In contrast, the ranking of other candidate genes in the six groups varied greatly. A similar phenomenon has been observed in *Ammopiptanthus mongolicus* [35]. *EIF1* and *EIF3* were selected as reference genes across all of the samples, whereas these two genes were the most stable only under drought stress among the four evaluated abiotic stresses. Following acibenzolar-*S*-methyl treatment, the combination of *CYP* and *eIF4B* was most suitable as an internal control in *Eucalyptus* L’Hér. In addition to *P. cocos* and *Eucalyptus* [38], *CYP* has been selected as an internal control for several animal cells. In human peripheral blood, *CYP* was a more suitable housekeeping gene than *β*-*Act* and *GAPDH* [39]. *CYP* was also recommended as one of the reference genes for neurons of the central nervous system [40] and in atopic human bronchial epithelial cells [41]. Moreover, *CYP* was considered to be an RNA normalization control in rats [42].

**Table 11:** Ranking order of the reference genes for all treatment conditions (Better-Good-Average)

Method	1	2	3	4	5	6	7	8	9	10	11	12	13	14
DeltaCT	*CYP*	*his3*-*1*	*GAPDH*	*RP*	*RPB1*-*2*	*RPB2*	*β*-*Act*	*his3*-*2*	*RPB1*-*1*	*MAPK*	*β*-*Tub*	*SAMDC*	*EIF*	*APT*
BestKeeper	*CYP*	*RPB1*-*2*	*β*-*Act*	*RP*	*his3*-*1*	*RPB1*-*1*	*GAPDH*	*RPB2*	*APT*	*MAPK*	*β*-*Tub*	*his3*-*2*	*SAMDC*	*EIF*
NormFinder	*CYP*	*his3*-*1*	*GAPDH*	*RP*	*RPB2*	*RPB1*-*2*	*β*-*Act*	*MAPK*	*his3*-*2*	*RPB1*-*1*	*β*-*Tub*	*SAMDC*	*EIF*	*APT*
geNorm	*his3*-*1/RP*		*CYP*	*β*-*Act*	*RPB1*-*1*	*RPB1*-*2*	*GAPDH*	*RPB2*	*MAPK*	*his3*-*2*	*β*-*Tub*	*SAMDC*	*EIF*	*APT*
Recommended comprehensive ranking	*CYP*	*his3*-*1*	*RP*	*RPB1*-*2*	*GAPDH*	*β*-*Act*	*RPB2*	*RPB1*-*1*	*MAPK*	*his3*-*2*	*β*-*Tub*	*SAMDC*	*APT*	*EIF*

NormFinder, BestKeeper and geNorm are widely used for selection of reference genes, although the results generated by the different methods may be slightly different [43, 44]. Our results displayed the same tendency as those of previous studies [26–30]. Moreover, the validity of the results might be related to the materials used or even to potential experimental errors. The importance of systematic evaluation before candidate genes are used as reference genes, especially under different conditions were observed in the study.

## Conclusion

*SAMDC*, *his3*-*2*, *RP*, *RPB2*, and *his3*-*1* were evaluated to be suitable reference genes for *P. Cocos* following different treatments. *RPB2* was the most stable reference gene under different temperatures and *CYP* was the most stable gene in the mycelia under all six evaluated conditions.

## Abbreviations

GAPDH: glyceraldehyde-3-phosphate dehydrogenase
MAPK: mitogen-activated protein kinase
β-Act: beta actin
RPB2: RNA polymerase subunit 2
RPB1-1: RNA polymerase subunit 1
RPB1-2: RNA polymerase subunit 1
his3-1: histone 3
his3-2: histone 3
APT: adenine phosphoribosyl transferase
SAMDC: *S*-adenosyl methionine decarboxylase
RP: ribosomal protein
β-Tub: beta tubulin
EIF: eukaryotic translation initiation factor
CYP: cyclophilin
IAA: indole-3-acetic acid
ABA: abscisic acid
6-BA: 6-benzylaminopurine
MeJA: methyl jasmonate
GA: gibberellin acid
qRT-PCR: quantitative real-time PCR
PCR: polymerase chain reaction
